# Emerging role of non-coding RNAs in resistance to platinum-based anti-cancer agents in lung cancer

**DOI:** 10.3389/fphar.2023.1105484

**Published:** 2023-01-26

**Authors:** Priya Mondal, Syed Musthapa Meeran

**Affiliations:** ^1^ Department of Biochemistry, CSIR-Central Food Technological Research Institute, Mysore, India; ^2^ Academy of Scientific and Innovative Research (AcSIR), Ghaziabad, India

**Keywords:** platinum drugs, platinum resistant cancer, non-coding RNAs, cisplain, carboplatin, chemoresistance, microRNA

## Abstract

Platinum-based drugs are the first line of therapeutics against many cancers, including lung cancer. Lung cancer is one of the leading causes of cancer-related death worldwide. Platinum-based agents target DNA and prevent replication, and transcription, leading to the inhibition of cell proliferation followed by cellular apoptosis. About twenty-three platinum-based drugs are under different stages of clinical trials, among cisplatin, carboplatin, and oxaliplatin are widely used for the treatment of various cancers. Among them, cisplatin is the most commonly used drug for cancer therapy, which binds with RNA, and hinders the cellular RNA process. However, long-term use of platinum-based drugs can cause different side effects and has been shown to develop chemoresistance, leading to poor clinical outcomes. Chemoresistance became an important challenge for cancer treatment. Platinum-based chemoresistance occurs due to the influence of intrinsic factors such as overexpression of multidrug resistance proteins, advancement of DNA repair mechanism, degradation, and deactivation of intracellular thiols. Recently, epigenetic modifications, especially non-coding RNAs (ncRNAs) mediated gene regulation, grasp the attention for reversing the sensitivity of platinum-based drugs due to their reversible nature without altering genome sequence. ncRNAs can also modulate the intrinsic and non-intrinsic mechanisms of resistance in lung cancer cells. Therefore, targeting ncRNAs could be an effective approach for developing novel therapeutics to overcome lung cancer chemoresistance. The current review article has discussed the role of ncRNA in chemoresistance and its underlying molecular mechanisms in human lung cancer.

## 1 Introduction

Globally lung cancer statistics reported an 18% mortality rate in 2020, which is responsible for more cancer-associated deaths worldwide. The American Cancer Society also reported the highest mortality rate due to lung cancer. According to the American Cancer Society, about 236,740 new cases of lung cancer (117,910 in men and 118,830 in women) and about 130,180 deaths (68,820 in men and 61,360 in women) have been estimated due to lung cancer in the United States alone in 2022 (Siegel et al., 2022). Most commonly, about 85% of lung cancer patients are, in general, diagnosed with non-small cell lung cancer (NSCLC), and the rest with small cell lung cancer (SCLC).

Chemotherapy is one of the most commonly used and primary treatment options for lung cancer. Chemotherapy is chosen because most patients (77%) are diagnosed at later stages when surgery for curative intent is ineffective. Therefore, chemotherapy became one of the basic options to determine the survival and quality of life of the patients. Platinum-based drugs are the backbone of anti-cancer therapeutic agents. Some popular platinum-based chemotherapeutics are cisplatin, carboplatin, oxaliplatin, nedaplatin, lobaplatin, and hepatoplatin. There are about 23 platinum-based drugs are under different stages of clinical trials. Among them, cisplatin, carboplatin and oxaliplatin are approved and widely used drugs for treating various cancers. Cisplatin is the first-generation platinum-based anti-cancer agent approved by the US FDA. Cisplatin is considered one of the first-line therapeutic agents for treating various cancers, such as ovarian, lung, breast, and colon cancers. Non-specific targeted therapy of cisplatin causes several side effects, thereby less toxic carboplatin-the second-generation drug, and oxaliplatin-the third-generation drug, were developed and approved globally for cancer treatment (Wheate et al., 2010). These three successful platinum-based anti-cancer drugs are still extensively used for cancer therapy.

Long-term use of these anticancerous agents have been associated with several side effects, including chemoresistance and followed by tumor relapse (Wheate et al., 2010). Recently, chemoresistance has become a vital challenge in chemotherapy. In chemoresistance, cancer cells can escape or survive over the effect of drugs by altering several cellular mechanisms such as tumor microenvironment, drug transporters, genetic and epigenetic factors. Among different epigenetic factors, non-coding RNAs (ncRNAs) functions as ‘the sword and the shield’ over chemoresistance (Mondal and Meeran, 2021). The aberrant expression of ncRNAs regulates various molecular mechanisms which lead to chemoresistance. At the same time, several ncRNAs reverse the resistance by targeting the intracellular factors involved in chemoresistance. Among different ncRNAs, microRNAs are the most active small ncRNAs (∼22 nucleotides in size) and play a significant role in the chemoresistance of platinum-based anti-cancer agents. This review summarises the recent scientific evidence on various intracellular resistance mechanisms involved in platinum-based anti-cancer agents. This review also discussed the emerging role of ncRNAs in reversing the chemoresistance of platinum-based anti-cancer agents.

## 2 Mode of action and limitation of platinum-based anti-cancer agents in lung cancer

Platinum-based doublet chemotherapy is the backbone for treating lung cancer, especially NSCLC. Generally, Chloride (Cl) concentration is relatively high (100 mM) in the blood stream, therefore, chloride ligands stay attached to the drug though it also binds to serum proteins, such as human serum albumin. But when it reaches the tumor site, the platinum-based agent enters the cells by mainly three pathways: passive diffusion, copper transporter proteins (CTR1) and/or organic cation transporters. Inside the cell, if the chloride concentration is decreased (4–20 mM), then platinum-based agents will be aquated with the loss of one or both of the chloride ligands [Pt(NH_3_)_2_Cl(OH_2_)]^+^ and [Pt(NH_3_)_2_(OH_2_)_2_]^2+^, and target DNA, thereby, prevent replication and transcription leading to cellular apoptosis (Wheate et al., 2010). In addition, cisplatin is also known to bind to RNA and hinders transcription. Due to its precise mechanism of action, cisplatin has become the first line of choice of Platinium-based drugs for treating various cancers. However, long-term use of cisplatin has been shown to cause different side effects, such as nephrotoxicity, and neurotoxicity, due to the off-target effects (Zhang et al., 2022). Therefore, the second-generation platinum-based drug carboplatin was developed to overcome the toxicity and improve the efficacy of cisplatin.

**FIGURE 1:**
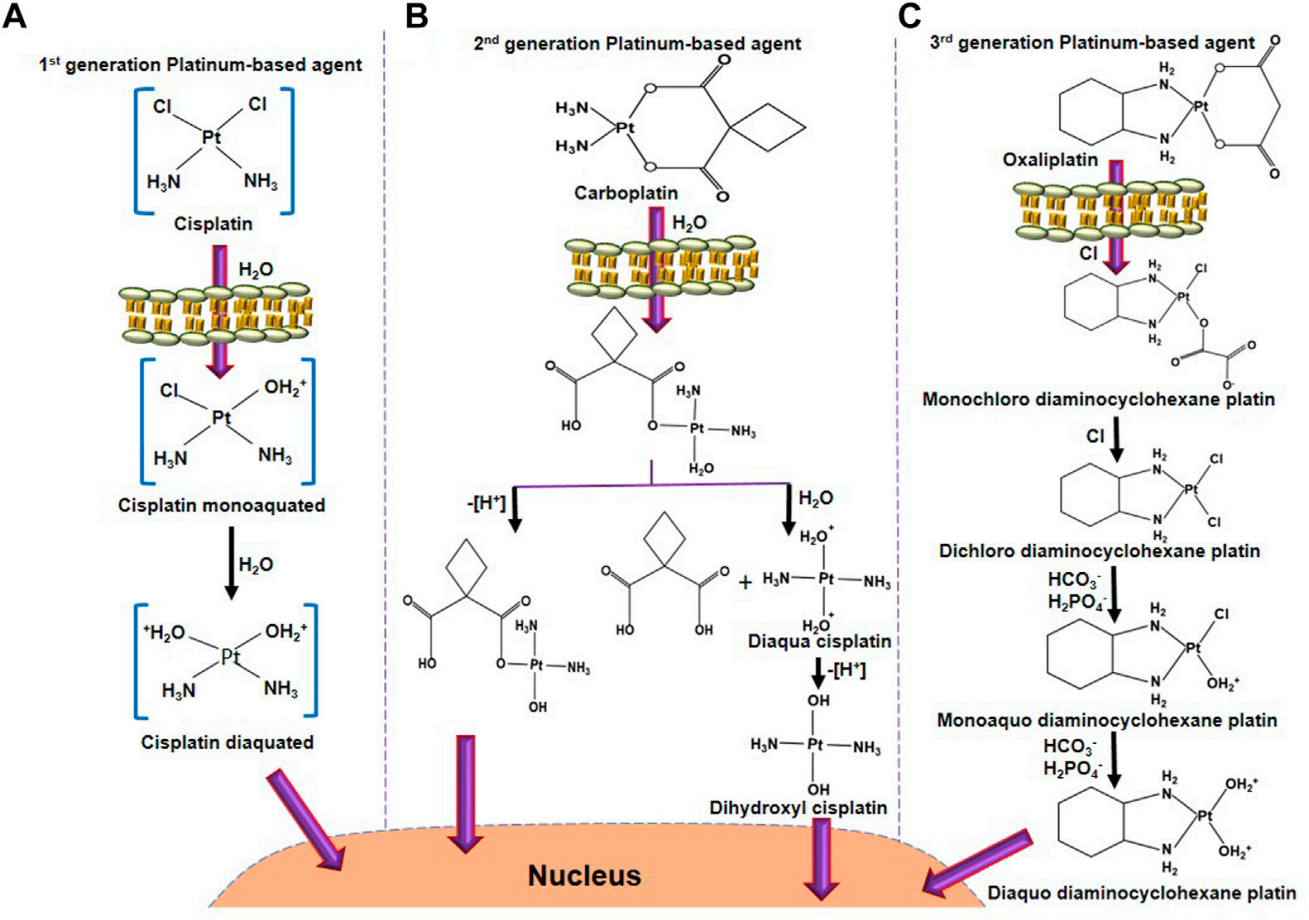
The activation process of platinum-based anticancer agents. **(A)** Cisplatin activation starts by exchanging one chloride molecule with a water molecule, forming cisplatin monoaquated after entering into the cell membrane. In the next step, after hydrolysis, another chloride molecule of cisplatin monoaquated was replaced to form cisplatin diaquated. This active form of cisplatin enters into the nucleus to form DNA adducts. **(B)** Carboplatin activation starts after hydrolysis, one bond between platinum and 1,1-cyclobutanedicarboxylate. One side oxidation will form a hydroxyl derivative of a mono-hydrolyzed carboplatin molecule. Another side, further hydrolysis will form diaqua cisplatin. Oxidation of diaqua cisplatin further forms dihydroxyl cisplatin, the active form of carboplatin. **(C)** After entering through the cell membrane, one oxygen molecule of oxaliplatin was exchanged with a chloride molecule to form monochloro diaminocyclohexane platin. A similar reaction occurs, leaving chloride molecules to form dichloro diaminocyclohexane platin. Further, interaction with HCO_3_^−^ and H_2_PO_4_^−^ forms monoaquo diaminocyclohexane platins. A similar bicarbonation and phosphorylation forms diaquo diaminocyclohexane platin, which is the active form of oxaliplatin. This active oxaliplatin derivative enters the nucleus to form DNA adducts.

Early clinical trials of carboplatin were performed in 1982, and in the year 1989, FDA approved this drug for therapeutic use. In the structure of cisplatin, two chloride atoms was substituted with an oxygenated bidendate cyclobutanedicarboxylate group through hydrolysis to form carboplatin as shown in Figure 1. Therefore, carboplatin becomes a positively charged molecule that covalently binds to the N7 site of purine bases of nucleophile molecules such as DNA, RNA, and protein. This covalent bond with the alkyl group to nucleotides leads to mono adducts that cause DNA fragmentation, thereby inducing apoptosis. Similarly, another second-generation platinum-based anti-cancer agent Nedaplatin [cis-diammine (glycolato-O^1^, O^2^) platinum] approved in Japan, which has almost 10 times more soluble than cisplatin and also causes less toxicity compared to cisplatin (Kuwahara et al., 2009; Zhong et al., 2018). On the other side, about 2% of carboplatin causes DNA cross-linking, where a base on one strand complements a base on another, thereby preventing DNA strands from transcription (Povirk and Shuker, 1994). This is the most cytotoxic action of carboplatin, generating errors in DNA replication and accumulating cells in the G_2_/M phase and causing programmed cell death (de Sousa et al., 2014).

Carboplatin also causes different mutations in cells. Carboplatin has a similar anti-cancer effect to cisplatin, however, cross-resistance with cisplatin has been observed in several cancers. The toxicity of carboplatin is lesser than cisplatin due to, firstly, bidendate cyclobutanedicarboxylate group being a poorer leaving group than chloride. Secondly, carboplatin forms a lower rate of the adduct with DNA (Hah et al., 2006). Third, carboplatin has shown a lower hydration rate due to the bidentatecyclobutane dicarboxylic acid ligands (Dilruba and Kalayda, 2016; Hu X. et al., 2017c). However, carboplatin loses its activity before reaching its target because carborplatin has a much faster reaction with ammonia for transplatin than cisplatin. Therefore, a higher dose of carboplatin can be used for cancer therapy, nevertheless, higher concentration for long periods of consumption leads to chemoresistance similar to cisplatin. Hence, third generation of platinum-based anticancer drug oxaliplatin {[oxalate (2-)-O, O′][1R,2R-cyclohexanediamine-N, N’] platinum-(II)} developed. In this structure, 1, 2-diaminocyclohexane (DACH) ligand alternatives for the amine groups of cisplatin. Yet, oxaliplatin generates less number of DNA adducts, but it causes higher cellular toxicity compared to cisplatin (Woynarowski et al., 2000). Oxaliplatin is strongly used for colon and gastrointestinal cancer, although cisplatin and carboplatin were ineffective against these cancers. The mode of action of oxaliplatin is similar to cisplatin in short of making any cross-resistance with cisplatin or carboplatin (Hu et al., 2019; Browning et al., 2017). Besides oxaliplatin, lobaplatin (cis-[trans-1,2-cyclobutanebis(methylamine)][(S)-lactato-O1, O2]platinum(II), another third-generation platinum-based anti-cancer drug, has been first permitted to use in China against breast cancer, leukemia, and NSCLC therapy (Wheate et al., 2010).

Generally, at the initial stage of cancer therapy, cancer patients respond very well to platinum-based agents, however, long-term use of these drugs leads to resistance. There are several mechanisms involved in resistance to platinum-based anti-cancer agents. Among them, three are well investigated in drug resistance: first, over activity of drug-efflux transporters, which reduced the concentration of chemotherapeutics inside the cells. Second, degradation and deactivation of intracellular thiols. Third, advanced intracellular DNA repair mechanism. Recently, it has been reported that the involvement of tumor microenvironment (TME), stem cells, and epigenetic modifications also play a key role in the regulation of platinum-based drug resistance (Mondal et al., 2021).

## 3 Non-coding RNAs on resistance to platinum-based anti-cancer agents

Non-coding RNAs (ncRNAs) have been known to regulate various biological processes such as cell cycle, cell proliferation, metastasis, and different cell signaling pathways. Aberrant regulation of ncRNAs has been reported in multiple diseases, including cancer chemoresistance (Mondal et al., 2020). The aberrant expression of ncRNAs can be reversed to enhance chemosensitivity by altering the expression of tumor-suppressor genes, tumor-promoter genes, and oncogenes. Many long non-coding RNAs (lncRNAs) are involved in cisplatin resistance to lung cancer. Recent studies have shown that ncRNAs, especially miRNAs, play a key role in the chemoresistance of platinum-based anti-cancer agents in lung cancer.

### 3.1 ncRNAs regulate resistance to platinum-based agents by altering the expression of drug transporters

Drug transporters play a key role in maintaining the cellular concentration of platinum-based anti-cancer agents. Overexpression of drug efflux transporters has been observed in cancer cells and this elevated expression of drug efflux transported enhances the efflux of chemotherapeutics from the cells into the outside. Therefore, these overexpressed drug efflux transporters help the cancer cells to evade the anticancerous effect, leads to chemoresistance. Generally, platinum-based agents enter the cells through passive diffusion and gated channels (Chen et al., 2015). However, some of the drug transporters such as ABCC1/multidrug resistance protein 1 (MRP1), ABCC2, ABCB9, MDR1/P-glycoprotein (P-gp) and lung-resistance protein (LRP) are also actively involved in platinum-based drug uptake or efflux. Therefore, aberrant expression of these drug transporters leads to chemoresistance to the platinum-based agents. Recent studies have shown ncRNAs can regulate the expression of these transporters, thereby altering the sensitivity of platinum-based agents in lung cancer cells (Table 1).

**TABLE 1:** Dysregulation of Non-coding RNAs on the drug transporters involved in cisplatin-resistance.

Non-coding RNAs	Dysregulation	Pathway/target	Sensitivity	Model	References
Let-7c	Downregulation	ABCC2 and Bcl-xL	Decrease	Cisplatin-resistant cells	Zhan et al. (2013)
miR-31	Upregulation	ABCB9	Decrease	NSCLC	Dong et al. (2014)
miR-21	Upregulation	MDR1, MPR1, GSH, E2F-1, Twist, PI3K/AKT signaling pathway	Decrease	A549/DDP	Dong et al. (2015)
XIST	Downregulation	MDR1 and MRP1	Increase	A549/DDP and H460/DDP cells	Tian et al. (2019)
miR-221	Downregulation	*PTEN* and pAkt	Increase	lung cancer cells	Wang et al. (2018b)

Among them, ABCC1, MDR1 and LRP are downregulated by miR-146a, which leads to the reversal of cisplatin sensitivity to cisplatin-resistant NSCLC cells (Shi L. et al., 2017a). Besides, miR-146a brings back cisplatin sensitivity in NSCLC cells by enhancing cell cycle arrest and programmed cell death through downregulating cyclin J and upregulation of the expression of cleaved caspase-3, respectively (Shi L. et al., 2017a). ABCC1 is also a target of miR-21, which involved in numerous ways of cisplatin resistance. For example, miR-21stimulates drug efflux by enhancing ABCC1 and P-gp expression. Moreover, miR-21 alters intracellular oxidative stress, hindering oxidative damage that impedes cisplatin-induced cell death. Because miR-21 increases the cystathionine and GSH level, superoxide dismutase and GST-π level, thereby indorsing drug inactivation.

Similar to ABCC1, ABCC2**,** a member of (ABC) transporters, effluxes intracellular molecules into extra-/intra-cellular space, thereby, cells become resistant to chemotherapeutics. On the other side, B-cell lymphoma-extralarge (Bcl-xL), a member of the Bcl-2 family, is a transmembrane anti-apoptotic protein preventing the release of mitochondrial proteins such as cytochrome c by making the mitochondrial membrane non-permeable and prevents program cell death. Overexpression of ABCC2 and Bcl-xL has been observed in cisplatin-resistant lung cancer (A549/DDP) cells. Also, reduced expression of Let-7c has been observed in A549 cells, which plays a key role in cisplatin resistance. Let-7c, a member of the let-7 family, directly binding to 3 ´UTR of *Bcl-XL* in cisplatin-resistant A549 cells and modulate the cisplatin sensitivity and induced apoptosis (Zhan et al., 2013). Hence, altering the expression of Let-7c can bring back the sensitivity of cisplatin in lung cancer cells by targeting ABCC2 and Bcl-xL (Zhan et al., 2013). In addition, bioinformatics analysis revealed that cell cycle regulators, such as *cyclin D1* and *STAT3,* are targets of let-7c, and overexpression of these genes leads to cisplatin resistance in lung cancer cells (Zhan et al., 2013). Similar to ABCC2, ATP-binding cassette, sub-family B (MDR/TAP), member 9 (ABCB9), another transporter of the ABC family associated with cellular transferring of chemotherapeutics and its allied MDR. miR-31 modulates the function of ABCB9, thereby altering cisplatin sensitivity (Dong et al., 2014). Upregulation of miR-31 represses cisplatin-induced cell death by modulating ABCB9 in lung cancer cells. Like miR-31, overexpression of miR27a and miR-451 also contribute to the MDR phenotype in cancer cells by regulating the expression of P-gp. Generally, overexpression of miR-27a and miR451 has been found in MDR-cancer cells compared to non-resistant cancer cells. Antagomirs of miR-27a or miR-451 reduced the expression of P-gp and MDR1, whereas the mimics of miR-27a and miR-451 enhanced their expression. The sensitivity of cytotoxicity has been enhanced when treated with antagomirs of miR-27a or miR-451. Therefore, targeting miR-27a and miR-451 can be a potential therapeutic strategy to regulate MDR in cancer cells (Zhu et al., 2008).

The interplay between lncRNA and miRNA alters cancer cell proliferation, tumor growth, and chemoresistance. Similar to miRNAs, lncRNAs also alter the expression of several drug transporters, such as MDR1 and MRPs. For example, lncRNA MALAT1 increases cisplatin resistance in lung cancer by enhancing the expression of MRP1 and MDR1 by activating the STAT3 pathway. Therefore, A549/MALAT1 resistant cells were highly resistant to the cisplatin-induced cell death program though A549/cisplatin/shMALAT1 cells are more prone to cisplatin-induced apoptosis (Fang et al., 2018). Similarly, XIST, another lncRNA involved in cisplatin resistance. Higher expression of XIST enhances cisplatin resistance by reducing the expression of miR-144-3p, as miR-144-3p is a target of XIST. Therefore, the knockdown of XIST inhibits growth and metastasis as well as enhances cell death by suppressing MDR1 and MRP1 expression in A549 and H460 cisplatin-resistant cells (Tian et al., 2019). Like XIST, another oncogenic lncRNA, ANRIL regulates cisplatin resistance by altering MRP1 and ABCC2 (Zhang D. et al., 2017a). ABCG2, another drug transporter target of SOX4, is a sex-determining region Y-box (SOX) family member. Both parental NSCLC cells and cisplatin-resistant NSCLC cells have shown upregulation of SOX4. SOX4 is a target of miR-130a-3p, which can inhibit the expression of SOX4. Therefore, the knockdown of SOX4 decreased miR-130a-3p-induced cisplatin resistance in cisplatin-resistant lung cancer cells by reducing ABCG2 expression (Hu B. et al., 2017a). On the other side, exogenous expression of SOX4 significantly abrogated the CCAT1-knockdown-mediated cisplatin sensitivity and ABCG2 lower expression in cisplatin-resistant lung cancer cells. Therefore, CCAT1/miR-130a-3p axis plays a crucial role in the cisplatin resistance of NSCLC cells by targeting SOX4 (Hu B. et al., 2017a).

### 3.2 ncRNAs alter the resistance to platinum-based agents by modulating the DNA repair pathways

**FIGURE 2:**
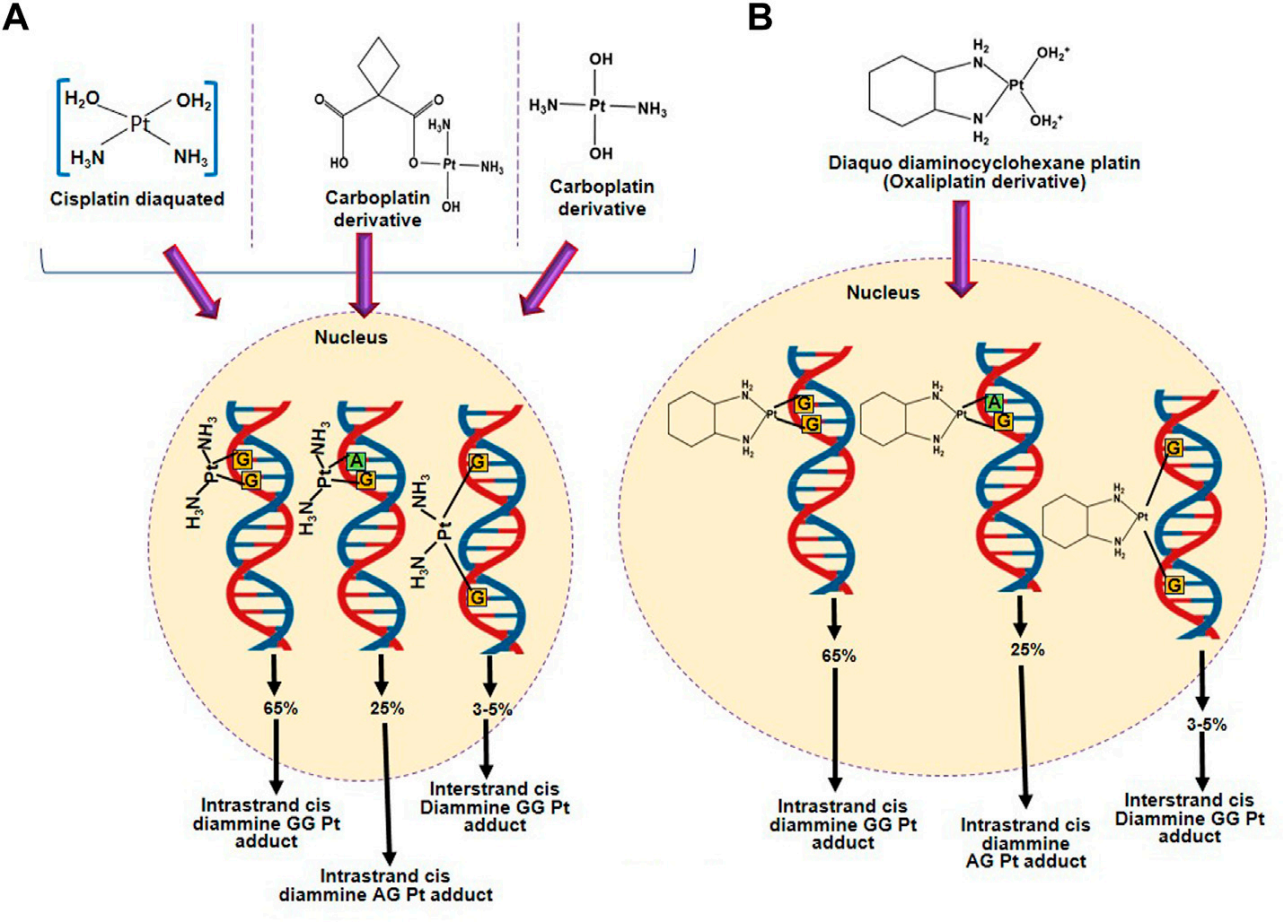
Formation of adducts between DNA and platinum-based agents. **(A)** DNA adduct formation with platinum-based anticancer agents having two amino groups (cisplatin and carboplatin). **(B)** DNA adduct formation with an active form of oxaliplatin.

Platinum-based agents form platinum-DNA adducts which regulate the cytotoxic effect of platinum-based agents by altering the double helical structure of DNA and nucleosomes, as shown in Figure 2 (Kartalou and Essigmann, 2001; Danford et al., 2005). Therefore, platinum-based agents hinder the replication and transcription and lead to DNA double-strand breaks (DSBs) after the initiation of the DNA repair mechanism. If the DNA damage exceeds the mean to repair, the cell will activate apoptosis to eliminate the cells. Therefore, the DNA repair mechanism is considered a crucial factor in resistance to platinum-based agents, except DNA mismatch repair, whose deficiency causes resistance to cisplatin/carboplatin (Zhou et al., 2020); Wynne et al., 2007; Sawant et al., 2015). Oxaliplatin is more cytotoxic than cisplatin as it initiates secondary DSBs and ribosomal biogenesis stress, whereas cisplatin causes more DNA damage (Bruno et al., 2017). The nucleotide-excision repair (NER) pathway removed the intra-strand crosslinks by eliminating the damaged nucleotides or generating DNA to rebuild genetic integrity (Roos and Kaina, 2013). Cisplatin is an alkyl agent that prevents DNA replication by the NER pathway. Furthermore, in the DNA repair mechanism, DNA repair enzymes such as excision repair cross-complementation group 1 (ERCC1) are involved in resistance to platinum-based agents.

ncRNAs regulate the platinum-based anti-cancer agent resistance by targeting the factors involved in the DNA repair mechanism. A study led by Wang *et al,* showed that 9 miRNAs are upregulated and another 5 miRNAs are downregulated in A549/cisplatin cells compared to parental A549 cells (Wang et al., 2011). Among them, the upregulation of miR-138 downregulates the expression of ERCC1 and increases the cisplatin sensitivity in A549/cisplatin cells by inducing apoptosis (Wang et al., 2011). ERCC1 is also regulated by miR-451 in ERCC1-highly expressed NSCLC cells. The upregulation of miR-451 majorly hinders the migration of ERCC1-highly expressed NSCLC cells by inhibiting Wnt/β-catenin and PI3K/AKT pathways. Simultaneously, upregulated miR-451 increases cisplatin sensitivity by reducing the expression of ERCC1 (Liu K et al., 2018b). LncRNA could serve as a competing endogenous RNA (ceRNA) of miRNA to modulate the expression of cancer-related target genes. Therefore, ectopic expression of lncRNA leads to the muddle of lncRNA/miRNA/mRNA network involved in cancer progression and chemoresistance. For example, overexpression of lncRNA Homo sapiens TatD DNase domain containing 1 (TATDN1) upregulates TRIM66 expression *via* sponging miR-451 and increases cisplatin resistance. Knockdown of TATDN1 enhanced cisplatin-sensitivity of NSCLC *via* blocking TRIM66 expression by competitive interaction with miR-451 (Wang L. et al., 2019a).

Differential expression of miRNAs is found in patients with lung adenocarcinoma (LAD). The hsa-miR-192, hsa-miR-1293, hsa-miR-194, and hsa-miR-561 are upregulated. Whereas hsa-miR-205, hsa-miR-30a, and hsa-miR-30c are downregulated (Hu et al., 2019). Overexpression of miR-192 significantly repressed apoptosis, enhanced proliferation, and conferred resistance to cisplatin in lung cancer cells by activating the NF-κB signaling pathway. Because NF-κB repressing factor (NKRF) is the direct target of miR-192. Further, miR-192 could promote cisplatin resistance by regulating G_1_/S transition and apoptosis by targeting NKRF. Therefore, targeting miR-192 could be a promising therapeutic strategy in cisplatin-resistance lung cancer (Li Y. et al., 2022b). miR-17 and miR-92 families play a key role in cisplatin resistance and can be considered as novel biomarkers for platinum-based chemotherapy in NSCLC (Zhang X. et al., 2018b). *RAS sarcoma (Ras)* have been reported as the most frequently activated oncogenes in various human cancer. NRAS proto-oncogene, one of the 3 members of the RAS oncogene family, stimulates the progression of tumorigenesis through the initiation of multiple downstream pathways such as mitogen-activated protein kinase 1 (MAPK)/extracellular signal-regulated kinase (ERK), phosphoinositide 3-kinase (PI3K)/AKT, and nuclear factor-kappa B (NF-kB) pathway. Interestingly, NRAS is a target of miR-29a and sensitizes the lung cancer cells to cisplatin. The overexpression of miR-29a enhanced the inhibition effects of cisplatin by targeting NRAS (Liu et al., 2018c). miR-29a also enhances cisplatin sensitivity in NSCLC by regulating the REV3-like DNA-directed polymerase zeta catalytic subunit (REV3L). REV3L is responsible for the translation of DNA and is also involved in the DNA repair pathway. Chen *et al* reported that the downregulation of REV3L or the upregulation of miR-29a inhibited cell growth, and improved the accumulation of cells in the G2/M phase of the cell cycle when co-treated with cisplatin, thereby reversing the cisplatin resistance (Chen et al., 2019). miR-29c, another member of the miR29 family, regulates cisplatin sensitivity to NSCLC cells by targeting the PI3K/Akt pathway (Sun D. M. et al., 2018a). Overexpression of miR-29c enhances cisplatin sensitivity by negatively regulating AKT2, a crucial factor of the PI3K/AKT pathway (Sun D. M. et al., 2018a). PI3K/AKT pathway is also a target of another miR-133b. The overexpression of microRNA-133b prevents cell proliferation and LDH activity, stimulates apoptosis and caspase-3 activity, represses the expression of EGFR, PI3K, p-Akt, p-JAK2 and p-STAT3 proteins, reduced cyclin D1 and increased Bax protein expression in cisplatin-induced A549 cells (Li et al., 2018). Similar to miRNAs, STAT3 proteins are also regulated by lncRNA, such as lncRNA HOMEOBOXA11 (HOXA11) antisense RNA (HOXA11-AS) increases cisplatin resistance in LAD cells *via* regulating the miR-454-3p-Stat3 axis. The overexpression of HOXA11-AS forecasts poor survival for LAD patients (Zhao et al., 2018).

LncRNA AK126698 induces cisplatin resistance to A549 cells by modulating NKD2 and FZD8, members of the Wnt signaling pathway (Yang et al., 2013). LncRNA AK126698 also decreases the degradation of β-catenin, and the knockdown of lncRNA AK126698 increases cisplatin resistance by regulating other signaling pathways such as TGF-β, ErbB along with the Wnt pathway (Yang et al., 2013). Similarly, MALAT1 knockdown cells have shown low expression of β-catenin and c-Myc, indicating that the Wnt signaling contributes to cisplatin resistance in NSCLC. SOX9, another member of the SOX family, is regulated by MALAT1 and hinders the action of miR-101. MALAT1 and miR-101 may compete for the same binding site of SOX9. SOX9 may bind the promoter site of MALAT1 and activates its transcription. By binding to SOX9, MALAT1 suppresses the expression of miR-101 and increases the expression of SOX9, which activates the Wnt signaling pathway. The feedback loop of SOX9, miR-101, and MALAT1 plays a critical role in the progression of chemoresistance by activating the Wnt signaling pathway. Therefore, there is an interlink between MALAT1-miR-101-SOX9 loops with WNT signaling in regulating cisplatin-related resistance (Chen et al., 2017). Like other signaling pathways, the MAPK/Slug signaling pathway is also involved in cisplatin resistance in lung cancer. LncRNA nicotinamide nucleotide transhydrogenase-antisense RNA1 (NNT-AS1) is highly expressed in drug-resistant NSCLC cells. LncRNA NNT-AS1 stimulates cisplatin resistance in NSCLC by regulating the MAPK/Slug signaling pathway (Cai et al., 2018).

### 3.3 ncRNAs regulate resistance to platinum-based agents through apoptotic pathways

**FIGURE 3:**
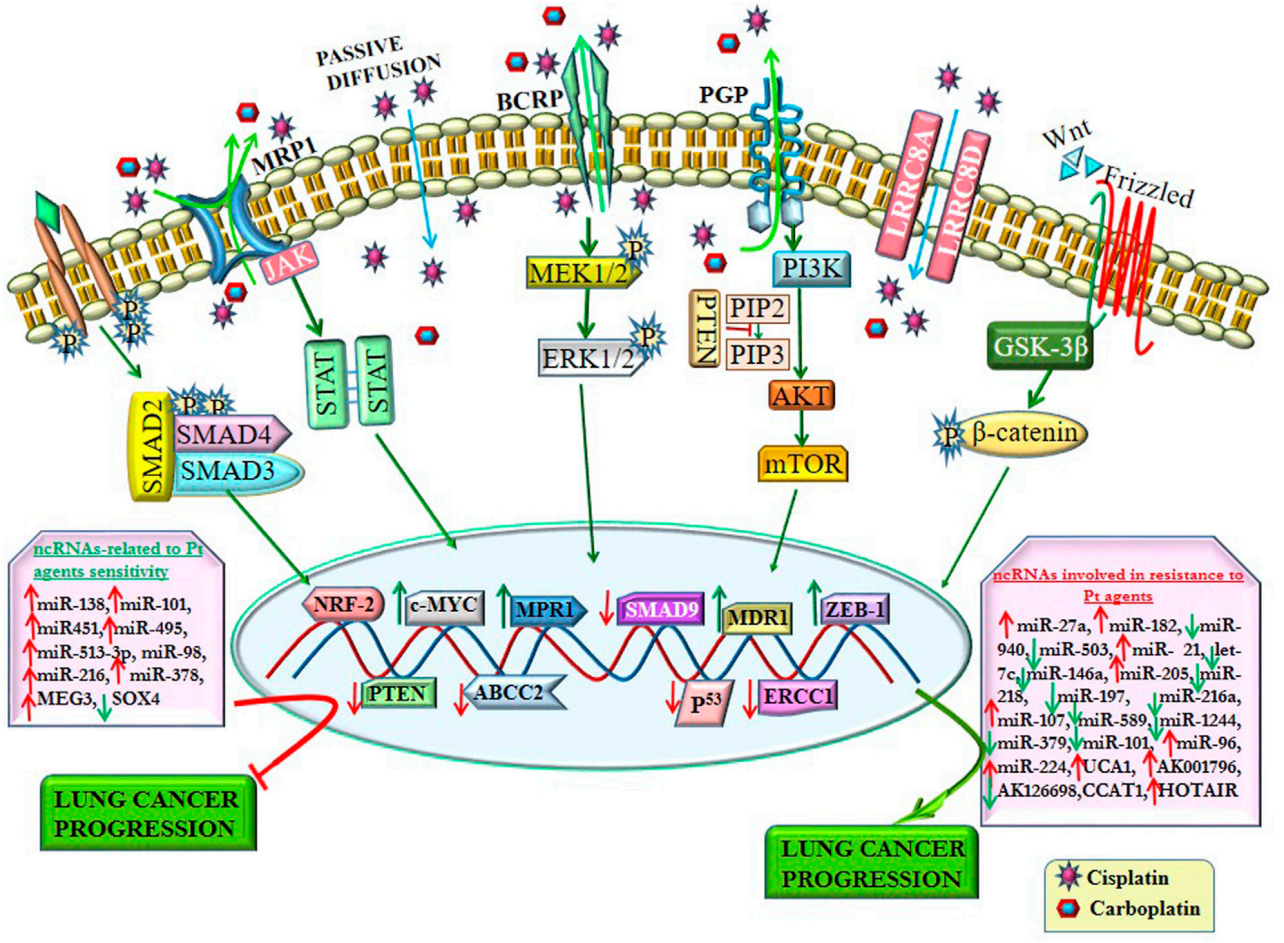
ncRNAs regulate resistance to platinum-based agents by altering signaling pathways and their related transcription factors. ncRNAs alter the drug efflux transporters (P-gp, MRP1, and BCRP) involved in chemoresistance by regulating various signaling pathways. Some ncRNAs enhance platinum-based agent (Pt)-resistance by altering the expression of several transcription factors. In contrast, some ncRNAs reverse the sensitivity of Pt by regulating different transcription factors, thereby impeding lung cancer progression.

Platinum-based agents prompt apoptosis by forming DNA adducts. Overexpression of anti-apoptotic proteins (BCL family) or defect in mitochondrial apoptotic pathways leads to resistance to platinum-based agents. Besides, several factors such as TME, signaling pathways (survival pathway and pathway of apoptosis), and epigenetic modifications, especially ncRNA regulation, are involved in resistance platinum-based agents, as shown in Table 2; Figure 3.

**TABLE 2:** Role of Non-coding RNAs on the regulation of signaling pathways involved in cisplatin-resistance.

Non-coding RNAs	Dysregulation	Pathway/target	Sensitivity	Model	References
miR-138	Upregulation	ERCC1	Increase	A549/DDP	Wang et al. (2011)
miR-451	Upregulation	ERCC1 Wnt/β-catenin and PI3K/AKT pathways	Increase	ERCC1-high NSCLC	Liu K et al. (2018b)
TATDN1	Downregulation	TRIM66 miR-451	Increase	NSCLC	Wang et al. (2019a)
AK126698	Downregulation	TGF-β, ErbB, Wnt pathway	Increase	Lung cancer	Yang et al. (2013)
miR-29a	Upregulation	REV3L	Increase	A549, H1650	Chen et al. (2019)
miR-29c	Upregulation	PI3K/Akt pathway	Increases	NSCLC	Sun et al. (2018a)
miR-133b	Upregulation	EGFR, PI3K, p-Akt, p-JAK2 and p-STAT3, Bax protein	Increases	A549 cells	Li et al. (2018)
HOXA11-AS	Upregulation	STAT3	Decrease	Lung adenocarcinoma	Zhao et al. (2018)
miR-494	Upregulation	CASP2	Decrease	NSCLC	Zhang et al. (2019)
MALAT1	Upregulation	Wnt signaling	Decrease	NSCLC	Chen et al. (2017)

Programmed cell death 4 (PDCD4), one of the major tumor suppressors involved in the cell death mechanism, has an inverse correlation with oncogene miR-182. The upregulation of miR-182 downregulates the expression of PDCD4, leading to increased cisplatin resistance (Ning et al., 2014). PDCD4 is another downstream target of miR-141. The downregulation of miR-141 could significantly upsurge cisplatin sensitivity and apoptosis of A549/cisplatin cells (Fu et al., 2016). In contrast, myeloid cell leukemia-1 (Mcl-1), a member of the anti-apoptotic Bcl-2 family, improves cell survival by preventing programmed cell death. Mcl-1 protein is a common factor behind the inverse correlation between these two ncRNAs. Mcl-1 is inversely regulated by miR-451 and by miR-135a/b in NSCLC. The upregulation of miR-451 brings back the cisplatin sensitivity to Mcl-1-knockdown A549/DPP cells and in cisplatin-resistant *in vivo* xenografts by downregulating Mcl-1 (Cheng et al., 2016). Similarly, Mcl-1 is inversely regulated by miR-135a/b. In A549/cisplatin cells, Mcl-1 is overexpressed, whereas the miR-135a/b is repressed compared to its parental A549. The downregulation of miR-135a/b in A549/cisplatin cells is mediated through epigenetic modification primarily by DNA methylation of CpG islands in the promoter region of *MIRN135A1*, *MIRN135A2*, and *MIRN135B* in cancer cells (Zhou et al., 2013). Therefore, it imposed miR-135a/b expression abridged Mcl-1 protein level and sensitized A549/cisplatin cells to cisplatin-induced apoptosis (Zhou et al., 2013). Mcl-1 is also regulated by lncRNA, such as MALAT1. MALAT1 regulates cisplatin-sensitivity in lung cancer cells by sequestering miR-101-3p from binding to 3′-UTR of *MCL1.* Therefore, the MALAT1/miR-101-3p/MCL1 signaling cascade plays a major role in cisplatin resistance in lung cancer. Knockdown of *MALAT1* and overexpression of miR-101-3p increase cisplatin sensitivity in lung cancer cells (Wang H. et al., 2018a).

Anti-apoptotic markers, Mcl-1 and Survivin, as well as pro-apoptotic proteins such as PARP, Caspase-3, and Bax are regulated by miR-205 and miR-218 in carboplatin-resistance. According to Zarogoulidis and his team, miRNAs have a ‘dual role’ as they can function as tumor enhancers or suppressors depending on the tumor microenvironment and external cellular stimuli. The upregulation of miR-218 can synergistically increase the efficacy of carboplatin in the lung cancer cell by activating the apoptotic pathway. In contrast, the upregulation of miR-205 increases resistance to carboplatin in LAD cells by hindering cellular apoptosis and boosting cancer cell to survive (Zarogoulidis et al., 2015). The mode of action of miRNAs are different, for example, Mcl-1 and Survivin have an inverse correlation with miR-218, whereas miR-205 targets PTEN and PHLPP2 to amplify the AKT signaling and drive malignant phenotypes in NSCLC. The upregulation of miR-218 decreases cell proliferation, invasion, and migration of lung cancer cells. miR-205 saved the oppressive effect of miR-218 by changing the expression levels of the pro-apoptotic proteins PARP and Caspase 3 (Zarogoulidis et al., 2015). Caspase-2 (encoded by *CASP2*), another member of the caspase family, contributes to cisplatin resistance. miR-494 promoted the proliferation and colony formation of NSCLC cells and reduced cisplatin-induced apoptosis by targeting CASP2 (Zhang et al., 2019).

Receptor-interacting protein 1 (RIP1) is a crucial adaptor kinase, controlling cell survival and cell death signaling pathways. RIP1 controls the function of different miRNAs. Among them, miR-940 is involved in cisplatin resistance. RIP1 represses JNK activation by releasing miR-940-mediated inhibition of Mitogen-Activated Protein Kinase Phosphatase 1 (MKP1) and suppressing the activation of Mitogen-Activated Protein Kinase Kinase 4 (MKK4) in cisplatin resistance. MKK4 is a member of the MAP kinase kinase family, which directly phosphorylates and activates the c-Jun NH2-terminal kinases (JNK) in response to cellular stresses and pro-inflammatory cytokines. The downregulation of RIP1 reduces MPK1 expression through miR-940 and finally blocks MKK4 and increases cisplatin resistance. Conversely, the lower expression of miR-940 increases MKP1 expression and reduces cisplatin-induced JNK activation and cell death. Similarly, RIP1-knockdown cells showed a higher expression of miR-940 and the lower expression of MKP1 (Wang Q. et al., 2014b).

The overexpression of tumor suppressor lncRNA maternally expressed gene 3 (MEG3) increases cisplatin sensitivity to cisplatin-resistant cells by inducing mitochondrial apoptosis pathway through Bcl-xl (Liu et al., 2015). The overexpression of MEG3 also enhanced cisplatin sensitivity in cisplatin-resistant lung cancer cells by regulating the miR-21-5p/SOX7 axis, *in vitro* and *in vivo* models, impeding cell proliferation, and inducing apoptosis (Wang et al., 2017). SOX7, a transcription factor belonging to the SRY-related HMG-box (SOX) family, regulates embryonic development and determines cell fate. miR-21-5p is a direct target point of SOX7. SOX7 has complementary binding sequences in 3 ´-UTR of miR-21-5p, whereas MEG3 directly interacted with miR-21-5p. MEG3 positively regulated SOX7 expression by suppressing miR-21-5p (Wang et al., 2017). In addition to miR-21-5p/SOX7, the downregulation of MEG3 increases cisplatin resistance of lung cancer cells *via* activation of the WNT/beta-catenin signaling pathway (Xia et al., 2015).

Upregulation of miR-503 suppresses the expression of anti-apoptotic protein Bcl-2 and increases cisplatin sensitivity and cisplatin-induced apoptosis in A549/cisplatin cells (Qiu et al., 2013). Bcl-2 is the target point of another miR-152. The downregulation of anti-apoptotic proteins Bcl-2 and NF-κB enhances cisplatin sensitivity among A549/cisplatin cells by upregulating miR-152 (Zhao et al., 2019). There is an inverse relationship between miR-630 and anti-apoptotic protein Bcl-2 detected by Kaplan–Meier, and Cox regression analysis, which has also shown poor survival rate and short relapse-free survival in NSCLC patients. The downregulation of miR-630 enhanced cisplatin resistance and colony formation by targeting Bcl-2 in NSCLC cells (Chen et al., 2018). Several ncRNAs target Bcl-2 family to alter the sensitivity of platinum-based drugs as shown in Table 3.

**TABLE 3:** Dysregulation of ncRNAs alters the expression of apoptotic markers involved in cisplatin-resistance.

Non-coding RNAs	Dysregulation	Pathway/target	Sensitivity	Model	References
miR-182	Upregulation	PDCD4	Decrease	A549 cells	Ning et al. (2014)
miR-141	Downregulation	PDCD4	Increase	A549/DDP cells	Fu et al. (2016)
miR-451	Upregulation	Mcl-1	Increase	A549/DPP cells cisplatin-resistant xenografts model	Cheng et al. (2016)
miR-135a/b	Downregulation	Mcl-1	decrease	A549/CDDP cells	Zhou et al. (2013)
MALAT1	Downregulation	Mcl-1	Increase	lung cancer cells	Wang et al. (2018a)
miR-101-3p	Upregulation	Mcl-1	Increase	lung cancer cells	Wang et al. (2018b)
miR-148b	Upregulation	DNMT1	Increase	A549/DDP	Sui et al. (2015)
miR-940	Downregulation	MKP1	Decrease	*In vitro* and *in vivo* model of lung cancer	Wang et al. (2014c)
Let-7c	Upregulation	Bcl-XL	Increase	A549/DDP cells	Zhan et al. (2013)
MEG3	Upregulation	p53, Bcl-XL	Increase	cisplatin resistant cells	Liu et al. (2015)
MEG3	Upregulation	SOX7	Increase	cisplatin resistant cells	Wang et al. (2017)
MEG3	Downregulation	miR-21-5p/SOX7	Decrease	lung cancer cells	Xia et al. (2015)
miR-503	Upregulation	Bcl-2	Increase	A549/DDP cells	Qiu et al. (2013)
miR-152	Upregulation	Bcl-2, NF-κB	Increase	A549/DDP cells	Zhao et al. (2019)
miR-630	Downregulation	Bcl-2	Decrease	NSCLC	Chen et al. (2018)
miR-184	Downregulation	Bcl-2	Decrease	E6-positive lung cancer patients	Tung et al. (2016)
miR-200bc/429 cluster	Upregulation	Bcl-2 and XIAP	Increase	lung cancer cells	Zhu et al. (2012)
LINC00485	Downregulation	CHEK1, Bcl-2, VEGF, HIF-1α, Bax	Increase	LAC cells	Zuo et al. (2019)
miR1236-3p	Upregulation	Pim-3 signaling pathway	Increase	A549/DDP cells	Wang et al. (2019c)
miR-155	Downregulation	Apaf-1-mediated pathway	Increase	A549 cells	Zang et al. (2012)
miR-218	Upregulation	apoptotic pathway	Increase	NSCLC	Zarogoulidis et al. (2015)
miR-205	Upregulation	PTEN and PHLPP2, AKT signaling	Decrease	lung adenocarcinoma cells	Zarogoulidis et al. (2015)
miR-197	Downregulation	PD-L1, CKS1B	Decrease	PD-L-1 positive patients	Fujita et al. (2015)

There are 4 members present in the miR-181 family such as miR-181a, miR-181c, and miR-181d. They play a strategic role in the chemoresistance of platinum-based agents. One of the ‘‘seed regions’’ i.e., 3′ UTR of *Bcl-2* is the target site of the mature miR-181s. Among them, miR-181b enhances cisplatin sensitivity in A549/cisplatin cells by inhibiting the expression of Bcl-2 (Zhu et al., 2010). Bcl-2 also de-targeted by E6-mediated miR-184 and thereby the reduction of Bcl-2 induces cisplatin resistance. miR-184 promoter activity and its expression were regulated by E6 and/or p53. p53 was bound onto the miR-184 promoter and its binding activity was also modulated by E6 and/or p53 manipulation. Patients with low-mR-184, E6-positive, high-Bcl-2 tumors, and both combinations were more prevalently did not show response to cisplatin-based chemotherapy than their counterparts (Tung et al., 2016). E6-positive lung cancer patients with lower expression of miR-184 and higher expression of Bcl-2 tumors or patients with E6-positive/low-miR-184, low-miR-184/high-Bcl-2, E6-positive/high-Bcl-2 tumors have shown less sensitivity towards cisplatin (Tung et al., 2016). 3 ´UTR of *Bcl-2* and another anti-apoptotic marker *X-linked inhibitor of apoptosis (XIAP)*, which prevents the action of certain enzymes such as caspase 3, 7, and 9, is the target point of miR-200bc/429 cluster. Zhu *et al* have demonstrated that miR-200bc/429 cluster is less expressed in A549/cisplatin cells compared with A549 cells. The overexpression of the miR-200bc/429 cluster increases cisplatin-induced apoptosis by repressing the expression of Bcl-2 and XIAP in lung cancer cells (Zhu et al., 2012). Bcl-2 is also a target point of HOXA transcript at the distal tip (HOTTIP), a newly identified lncRNA, sponging miR-216a and amplifying the expression of Bcl-2. *Bcl-2* is also an important target gene of miR-216a, and thereby, jointly enhanced chemoresistance of SCLC by regulating Bcl-2 (Sun Y. et al., 2018b).

DNA damage is the main reason for apoptosis, and checkpoint kinase 1 (CHEK1) is associated with DNA damage modification and identification. LINC00485 acted as a competitive endogenous RNA against miR-195, and miR-195 directly targeted CHEK1. Silencing of LINC00485 or overexpression of miR-195 increases cisplatin sensitivity by reducing the expression of CHEK1, Bcl-2, VEGF, and HIF-1α endorsing the apoptosis of LAC cells by increasing the expression of Bax (Zuo et al., 2019). Pim-3 is a proto-oncogene that prevents apoptosis and promotes cell survival through the phosphorylation of BAD, which prompts the release of Bcl-XL. The upregulation of miR1236-3p could reverse cisplatin resistance by inhibiting the Pim-3 signaling pathway in lung cancer cells (Wang Z. et al., 2019c). Another way to induce apoptosis is by destabilizing mitochondrial integrity and activating the intrinsic apoptotic pathway. Apoptotic protease activating factor 1 (Apaf-1) can bind with cytochrome c released from the mitochondrial inter-membrane and activate the initiator caspase-9, ultimately ensuing in cellular apoptosis. The downregulation of miR-155 can increase the cisplatin sensitivity to A549 cells by regulating cellular apoptosis and DNA damage through an Apaf-1-mediated pathway (Zang et al., 2012). Another transcriptor involved in apoptosis is CHOP (DNA damage-inducible transcript 3), which produces endoplasmic reticulum stress. CHOP and miR-146a have an inverse correlation, and miR-146a controls CHOP expression by targeting the 3′UTR region of *CHOP* in lung cancer cells (Tan et al., 2018). Clinical samples of patients with advanced NSCLC have shown overexpression of miR-125b causes noticeable inhibition of cisplatin-induced apoptosis and consequently increases the resistance to cisplatin (Cui et al., 2013).

### 3.4 ncRNAs modulate resistance to platinum-based agents by controlling cell division

Cell fate depends on the regulation of different genes and transcription factors*.* As we discussed that by forming platinum-DNA adducts, platinum-based agents trigger anti-tumor activity. In addition, the intra-strand of these platinum-DNA adducts is repaired by base excision and NER pathway during the G1 phase (Slyskova et al., 2018). Rajal et al. (2021) have shown at the time of cisplatin exposure, lung cancer proliferative cells were accumulated in the late G1/early S phase. They could sufficiently repair their DNA over multiple generations and rounds of replications. Therefore, this result signifies the underlying non-genetic and recovery mechanism of cisplatin chemoresistance. ncRNA regulates several cell division-related factors and signaling pathways, thereby reverse back the sensitivity of platinum-based agents in lung cancer cells, as shown in Table 4. Insulin-like growth factor 2 (IGF-2), a novel oncogene, has been reported to regulate cell divisions and apoptosis. LncRNA LUCAT1 regulates the cell cycle, apoptosis of NSCLC cells, and the resistance to cisplatin through targeting IGF-2. The upregulation of lncRNA LUCAT1 directly upregulates IGF-2 in A549/cisplatin cells (Wang W. et al., 2019b).

**TABLE 4:** Non-coding RNAs profile dysregulation alter the factors involved in cell cycle distribution.

Drug	Non-coding RNAs	Dysregulation	Pathway/target	Sensitivity	Model	References
Cisplatin	miR-21	Upregulation	PTEN	Decrease	NSCLC	Yang et al. (2015)
Cisplatin	miR-130b	Downregulation	Wnt/β-catenin pathway	Increase	A549 cells	Zhang et al. (2018a)
Cisplatin	miR-181	Upregulation	PTEN/PI3K/AKT/mTOR pathway, LC3 and ATG5	Increase	A549/DDP	Liu et al. (2018a)
Cisplatin	miR-451	Upregulation	Akt signaling pathway	Increase	*in vitro* and *in vivo* model	Bian et al. (2011)
Cisplatin	miR-1244	Downregulation	*p53*	Decrease	A549/DDP cell	Li et al. (2016)
Cisplatin	TRPM2-AS	Downregulation	p53 and p66^shc^	Increases	A549/DDP	Ma et al. (2017)
Cisplatin	miR-224	Upregulation	p21WAF1/CIP1-pRb pathway	Decrease	A549/DDP	Wang et al. (2014a)
Cisplatin	HOTAIR	Upregulation	p21WAF1/CIP1	Decrease	*in vitro* and *in vivo* model of NSCLC SCLC	Fang et al. (2016)
Cisplatin	miR1236-3p	Upregulation	*TPT1*	Decrease	Lung cancer	Wang et al. (2019c)
Cisplatin	miR-106b-5p	Upregulation	P-gp, *PKD2*	Increase	Lung cancer	Yu et al. (2017)
Cisplatin	AK001796	Downregulation	CCNC, BIRC5	Increase	A549	Liu et al. (2017)

*PTEN* is a negative regulator of the PI3K/AKT signaling pathway and functions as a tumor suppressor gene regulating the cell cycle, apoptosis, and growth of solid tumors, including NSCLC. NF-κB/PTEN is modulated by miR-21 and sensitizes NSCLC to cisplatin. miR-21 reduces the expression of *PTEN* by complementary binding to 3 ´ UTR of this gene and induces cisplatin resistance. NF-κB regulates the expression of miR-21 by binding to the promoter site of *miR-21*. Therefore, NF-κB-induced miR-21 decreases the expression of *PTEN* and increases the cisplatin resistance in NSCLC (Yang et al., 2015). Thus, miR-21 is one of the crucial factors responsible for cisplatin resistance in lung cancer. *PTEN* is another major target of miR-130b to persuade resistance to cisplatin in lung cancer cells by triggering the Wnt/β-catenin signaling pathway (Zhang Q. et al., 2018a). The downregulation of miR-130b reversed cisplatin sensitivity and reduced growth, enhancing the apoptosis of A549 cells *via* the Wnt/β-catenin pathway (Zhang Q. et al., 2018a). miR-181c leads to cisplatin resistance in NSCLC cells by targeting Wnt inhibitory factor 1 (WIF1). WIF1 binds to Wnt proteins and prevents the Wnt/β-catenin signaling cascade (Zhang H. et al., 2017b). miR-181c directly targeted WIF1 and thereby activating the Wnt/β-catenin pathway through inhibiting WIF1 activity in A549/DDP cells. Simultaneously, by activating the Wnt/β-catenin pathway, miR-181c reduced the sensitivity of cisplatin in A549 cells. Therefore, inhibition of miR-181c enhanced cisplatin sensitivity in DDP-resistant NSCLC cells and also repressed the Wnt/β-catenin signaling cascade by targeting WIF1 (Zhang H. et al., 2017b).

In contrast to miR-21, overexpression of tumor suppressor miR-451 reduces cell growth and increases cisplatin sensitivity *in vitro* and *in vivo* by inactivating the Akt signaling pathway (Bian et al., 2011). The downregulation of tumor suppressor *p53* by miR-146a increases cisplatin resistance towards cisplatin (Shi L. et al., 2017a). Similar to miR-146a, *p53* is another target for miR-1244. The lower expression of miR-1244 eliminates the reduced expression of p53 at both gene and protein levels. Downregulation of miR-1244 in A549/cisplatin-resistant cells has been shown to develop cisplatin chemoresistance by inhibiting p53*-*regulated cell signaling pathways (Li et al., 2016). Transient receptor potential cation channel subfamily M member 2 (TRPM2) is involved in numerous processes that involve signaling *via* intracellular Ca^2+^ level. LncRNA TRPM2-AS, an antisense of TRPM2, regulates SHC1 and is an adaptor protein containing three isoforms such as p46, p52, and p66. LncRNA TRPM2-AS is highly expressed in A549/cisplatin cells compared to parental A549 cells. Knockdown of lncRNA TRPM2-AS increases cisplatin sensitivity by inducing cell apoptosis and alteration of cell cycle distribution through activation of p53 and p66^shc^ in A549/cisplatin cells (Ma et al., 2017). Alike miR-1224, the downregulation of miR-589 increases cisplatin resistance in NSCLC. In contrast, the upregulation of a few miRNAs, such as miR-495, miR-513a-3p, and miR-98, increase cisplatin sensitivity (Li et al., 2016).

Like apoptotic proteins, cell cycle-related proteins play a significant role in chemoresistance. Zhe Zhang *et al* showed that the tumor suppressor miR-107 is associated with cisplatin resistance by regulating the expression of cyclin-dependent protein kinase 8 (CDK8) in NSCLC A549 cell lines. The expression of miR-107 and CDK8 always have a negative correlation. The lower expression of CDK8 increases cisplatin sensitivity in NSCLC (Zhang et al., 2014). Similar to cyclin-dependent kinase proteins, cyclin-dependent kinase inhibitors are also regulated by miRNAs. For example, lower expression of miR-17 and miR-92 families regulates cyclin-dependent kinase inhibitor 1A (CDKN1A) and RAD21 cohesin complex component (RAD21) in cisplatin-resistant NSCLC (Zhao J. et al., 2015a). CDKN1A, also known as p21, controls the cell cycle and RAD21 is a double-strand-break repair protein. Overexpression of CDKN1A and RAD21 inhibits DNA synthesis and the repair of DNA damage mechanism, thereby, these two factors play a crucial role in cisplatin resistance.

p21WAF1/CIP1, a dominant cyclin-dependent kinase (CDK) inhibitor, hinders the activity of cyclin-CDK2 or cyclin-CDK4 complexes and prevents DNA replication by binding to proliferating cell nuclear antigen (PCNA). The Cip/Kip family members, specifically p21Waf1/CIP1, are responsible for cell cycle control, blocking the transition from phase G1 to phase S (Meeran and Katiyar, 2008). Besides being an inhibitor of cell cycle progression, p21WAF1/CIP1 also involves in the apoptosis pathway (Abbas and Dutta, 2009). p21WAF1/CIP1 is an efficient target point of miR-224. The upregulation of miR-224 significantly reduces the sensitivity of cisplatin to A549/cisplatin cells by regulating the p21WAF1/CIP1-pRb pathway and the intrinsic mitochondrial apoptotic pathway. These actions disturb the G1/S transition of the cell cycle and apoptosis in LAD cells (Wang H. et al., 2014a). Therefore, dysregulation of p21WAF1 (p21) plays a major role in cisplatin resistance *in vitro* and *in vivo* models of lung cancer.

LncRNA Homeobox (HOX) transcript antisense RNA (HOTAIR) is associated with the human HOX loci and has an inverse correlation with p21WAF1. HOTAIR is proficient in chromatin remodeling and stimulating cancer cell metastasis. Zhili Liu *et al* reported, for the first time, that there is a correlation between HOTAIR expression and tumor chemoresistance (Liu et al., 2013). It is proved that overexpression of HOTAIR reduces the sensitivity of cisplatin in NSCLC. siRNA/HOTAIR1-mediated chemosensitivity is enhanced by inhibiting cell proliferation and induction of the G_0_/G_1_ cell-cycle arrest and also causes apoptosis by modulating p21WAF1/CIP1 expression. HOTAIR can also regulate chemoresistance in SCLC. Like NSCLC, the downregulation of HOTAIR also induced chemosensitivity in SCLC by increasing cellular apoptosis and suppressing tumor development *in vivo* (Fang et al., 2016).

LncRNA HOTAIR can modulate chemoresistance in SCLC by DNA methylation of HOXA1. HOXA1, one of the HOX family members, has been reported to be involved in many important biological functions, including cell growth, apoptosis, transformation, and survival. Translationally controlled tumor protein 1 (TPT1) is a key regulator of cellular growth and proliferation. This protein also controls a variety of cellular pathways involved in cell division and apoptosis. This protein plays a major role in carcinogenesis and chemoresistance. Wang *et al* showed that the *TPT1* is a direct target of miR-1236-3p by a dual-luciferase assay. The overexpression of miR1236-3p could reverse cisplatin resistance by targeting *TPT1* in lung cancer cells (Wang Z. et al., 2019c).

Similar to miRNAs, lncRNAs also have a major role in regulating cisplatin resistance in NSCLC. LncRNAs UCA1 has shown higher expression in cisplatin-resistant NSCLC than parental NSCLC. The overexpression of lncRNAs UCA1 reduces cisplatin sensitivity in both A549 and H1299 cells (Hu L. et al., 2017b). Alike lncRNAs UCA1, A549/cisplatin cells also have shown higher expression of lncRNA AK001796 than parental A549 cells. Knockdown of lncRNA AK001796 induces sensitivity of cisplatin by enhancing the expression of factors involved in cell cycle and apoptosis such as cell cycle proteins cyclin C (CCNC), baculoviral IAP repeat containing 5 (BIRC5), and also by suppressing the expression of cell cycle-associated (Liu et al., 2017).

### 3.5 ncRNAs alter resistance to platinum-based agents by regulating the epigenetic modifications

Epigenetic modifications such as DNA methylation, histone modification and ncRNA-regulated gene expression promote cancer progression by altering the nucleosomal assembly and chromosomal stability. Moreover, promoter hypermethylation of TSGs enhances cancer progression by suppressing the expression of TSGs. Alternatively, aberrant global DNA hypomethylation causes genome instability and activates the silenced genomic region as well as oncogenes. Similarly, histone modification is another key epigenetic modification, which alters the structure of chromatin through methylation, acetylation, phosphorylation, ubiquitination, and sumoylation of amino acid residues present in the histone tails. These modifications alter the accessibility of DNA to the various key transcriptional factors involved in gene expression. Therefore, inappropriate histone modification plays a crucial role in driving the progression of chemoresistance, followed by carcinogenesis. ncRNAs, especially miRNAs, modulate several cellular activities, such as cell proliferation, migration, and apoptosis, by altering the expression of TSGs and oncogenes. Hence, the upregulation of oncomiRNAs and downregulation of tumor suppressor miRNA is also associated with cancer progression. Recently, several studies have shown that these epigenetic modifications are also involved in chemotherapy and chemoresistance mechanisms. Interaction between these epigenetic regulations alters the chemosensitivity of many drugs, including platinum-based anti-cancer agents. For example, DNA methyltransferases (DNMTs) are functional epigenetic enzymes that are involved in DNA methylation and associated with epigenetic processes related to chemotherapy (Sui et al., 2015). miR-148b controls the expression of DNMT1 by targeting 3′ untranslated region (3′UTR) of *DNMT1* in cisplatin-resistance of NSCLC cells. miR-148b has inverse correlation with DNMT1 in the A549/cisplatin cells. DNMT1 was found to be upregulated in A549/cisplatin-resistant cells. The overexpression of miR-148b enhanced cisplatin sensitivity to A549/cisplatin cells through suppressing the expression of DNMT1 (Sui et al., 2015).

### 3.6 ncRNAs alter resistance to platinum-based agents by regulating the tumor microenvironment

The tumor microenvironment (TME) is an important factor in cancer progression and therapeutics. Effective therapeutic responses depend on the interaction between tumor cells and their surrounding microenvironments. Similarly, the interaction between tumor cells and stromal cells also influenced the mechanism of chemoresistance (Mondal et al., 2021). The imbalance of soluble factors, including growth factors, chemokines, and cytokines, alters the TME. Tumor-associated macrophages (TAMs) play a crucial role in TME by secreting anti-inflammatory cytokines, which contribute to tumor growth and an immunosuppressive environment. TAMs hinder the cytotoxicity of NK cells and enhance the release of programmed cell death ligand 1 (PD-L1), which prevents the anti-cancer activity of T cells. Recently, in acquired DDP-resistant NSCLC, PD-L1 expression was found to be enhanced. Furthermore, the combination of DDP and PD-L1 blockage can improve the prognosis of NSCLC patients (Wu et al., 2019). This indicates that increased PD-L1 expression might contribute to acquired DDP resistance and altering the TME in NSCLC. Overexpression of miR-181a was observed to regulate c-FOS expression in DDP-treated NSCLC. c-FOS is a subunit of transcription factor AP-1, which can directly bind the first intron of the PD-L1 gene (Green et al., 2012). Consistently, exogenous c-FOS in NSCLC could further elevate DDP-induced PD-L1 expression in NSCLC. Thus, DDP may regulate PD-L1 expression through c-FOS and miR-181a in NSCLC. In addition, cisplatin was found to induce negative feedback regulation of PD-L1 expression in NSCLC *via* ATM phosphorylation-mediated miR-181a expression. Furthermore, DDP-resistant NSCLC blocked this negative regulation to induce PD-L1 expression further. Overexpressed miR-181a in lung cancer could enhance the anti-tumor response *in vivo* by downregulating PD-L1 (Chen et al., 2022). In cancer patients, the primary TMEs contain ample inflammatory cytokines and macrophages expressing both the M1 and M2 phenotypes. Inflammasomes consist of a NOD-like receptor scaffold (NLR), adapter proteins, and caspase-1, which play a major role in innate immunity. NLRP3 inflammasome was found to be relatively high in cancer patients. On the other side, exosomal miR-21 promotes M2 polarization, and miR-21-containing tumor-secreted exosomes are potent regulators of TAMs by promoting M2-like activation. miR-21 modulates TME by NLRP3 phosphorylation and lysine 63-ubiquitination to inhibit the assembly of NLRP3 inflammasomes, which influences the post-chemotherapy TME and thereby reducing the chemotherapy response (Cheng et al., 2022).

Besides, blood flow, especially sufficient nutrients and oxygen to each cancer cell differ in solid tumors due to the presence of bulk cancer cells. This lesser blood flow to cancer cells leads to hypoxia and acclimatizes a survival strategy known as ‘autophagy’ to maintain cellular homeostasis by reducing the nutrient requirement. miRNAs play a significant role in regulating chemoresistance by targeting factors involved in TME. p62 is a multi-functional protein involved in autophagy by binding to LC3. Overexpression of p62 enhanced the aggregation of ubiquitinated proteins, thereby increasing cell survival strategy and cisplatin resistance. miR-199a-5p degraded p62 by directly binding to p62 mRNA in autophagy repressive and multidrug resistance H446/EP cells. Lower expression of miR-199a-5p increased p62, thereby promoting the transformation of LC3I to LC3II. Therefore, regulation of p62-mediated autophagy by miR-199a-5p was found to be a potential mechanism of SCLC cisplatin resistance (Li T. et al., 2022a). Uncoordinated-51-like kinase 1 (ULK1), an autophagy-related gene, is involved in the chemoresistance of diverse human cancers like liver, breast, and colon cancer. The LncRNA role of lung cancer-associated transcript 1 (LUCAT1) altered the sensitivity of cisplatin in NSCLC cells by upregulating ULK1 *via* sponging miR-514a-3p. LUCAT1 level has been observed to be higher in DDP-resistant NSCLC cells. Therefore, the knockdown of LUCAT1 improved the cisplatin sensitivity through inducing cell death and repressing autophagy and cell metastasis in NSCLC. Hence, the novel regulatory network of LUCAT1/miR-514a-3p/ULK1 plays a key role in cisplatin resistance in NSCLC (Shen et al., 2020). Autophagy-related protein 4D (ATG4D) and mTOR play a key role in autophagosome formation. *ATG4D* is also a target of miR-101-3p in NSCLC cells; thereby, inhibition of ATG4D by miR-101-3p inhibitor reverses the sensitivity of cisplatin in NSCLC cells. miR-101-3p mimic also enhances the sensitivity of cisplatin *in vivo* lung cancer models (Cui et al., 2021).

### 3.7 ncRNAs alter resistance to platinum-based agents by regulating EMT biomarkers

Epithelial-Mesenchymal Transition (EMT) is an advanced biological phenomenon where epithelial cells gradually acquire the features of mesenchymal (fibroblast-like) cells, thereby initiating the process of invasion and metastasis. Studies have shown that EMT is relevant in the progression of resistance toward platinum-based agents in lung cancer. Some major EMT markers are Snail, Slug, ZEB1, ZEB2, and Twist1. EMT-allied with various signaling pathways such as the transforming growth factor-β (TGFβ), Wnt/β-catenin, Hedgehog pathways play a major role in cisplatin and carboplatin chemoresistance. ncRNAs alter these EMT markers to regulate the metastatic nature of cancer cells (Mondal and Meeran, 2020). Here, we depicted the role of ncRNAs on the regulation of EMT markers which control the platinum-based drug sensitivity in cancer cells (Table 5). The TGFβ superfamily also plays crucial roles in cell proliferation, apoptosis, invasion, and metastasis. There are two types of cell-surface receptors, TGFβ receptor types 1 and 2 (TGFβ R1 and TGFβ R2), which are mainly involved in the transmission of TGFβ signaling to perform multiple intracellular functions. The miR-17 family (including miR-17, 20a, 20b) can repress TGFβR2, a core receptor of the TGFβ signal pathway, and reverse cisplatin-resistance in NSCLC (Jiang et al., 2014). Sterile α motif domain (SMAD) proteins function as signal transducers of TGF-β1 pathway and control cell growth, proliferation, differentiation, and apoptosis. TGF-β1-induced EMT of NSCLC cells enhanced the resistance to both erlotinib and cisplatin. Based on the literature, Ahmad *et al.* have discussed a correlation between EMT, drug resistance, Hedgehog signaling (Hh signaling), and the TGF-β1 signaling pathway (Ahmad et al., 2013).

**TABLE 5:** Non-coding RNAs profile dysregulation alter the potential targets involved in metastasis and chemoresistance.

Drug	Non-coding RNAs	Dysregulation	Pathway/target	Sensitivity	Model	References
Cisplatin	miR-96	Downregulation	FOXO3	Increase	Lung cancer	Wu et al. (2016)
Cisplatin	miR-216a	Upregulation	eIF4B and ZEB1	Increase	Lung cancer	Wang et al. (2014b)
Carboplatin	miR-21	Upregulation	SMAD7, TGFβ	Decrease	NSCLC	Lin et al. (2016)
Cisplatin	miR-449a	Upregulation	NOTCH1 signaling pathway	Increase	Lung cancer	You et al. (2014)
Cisplatin	miR-101-3p	Downregulation	SNHG1, ROCK2	Decrease	NSCLC	Wei et al. (2019)
Cisplatin	miR-218	Upregulation	RUNX2	Increase	NSCLC	Xie et al. (2016)
Cisplatin	miR-146a	Upregulation	Slug, ZEB2	Increase	NSCLC	Shi et al. (2017a)
Cisplatin	miR-27a	Upregulation	RKIP	Decrease	NSCLC, samples of patients	Li et al. (2014)

miRNAs target different signal transducer proteins, such as SAMD9 and SAMD7, involved in the TGF-β1 pathway, which are associated with EMT and play a major role in cisplatin resistance. In addition to Bcl-2, miR-181b also targets the 3′ -UTR of *TGFβ R1* and modulates the SMAD-dependent and the SMAD-independent TGF-β signaling by repressing TGFβR1. Knockdown of miR-181b increased cell proliferation, and metastasis of NSCLC by increasing the expression of p-SMAD3 (Wang et al., 2015). SAMD9, another transcription factor of the SMAD family, is negatively regulated by miR-96 and promotes chemoresistance in NSCLC. The downregulation of miR-96 upregulated SAMD9 which accelerates the action of cisplatin and suppresses lung cancer progression by regulating forkhead box O3 (FOXO3) (Wu et al., 2016). Wang *et al* also have shown that there is no significant relationship between miR-216a and SMAD7 in NSCLC, as observed in liver cancer (Wang R. T. et al., 2014c). Furthermore, they have demonstrated that miR-216a has an inverse correlation with metastasis markers such as eIF4B and ZEB1. The overexpression of miR-216a increases cisplatin-induced sensitivity and apoptosis by downregulating oncogenes like markers eIF4B and ZEB1. Clinical data have shown that patients with high levels of eIF4B and ZEB1 expression have a poor survival rate (Wang R. T. et al., 2014c). Similar to cisplatin resistance, SAMD7 has a crucial role in carboplatin resistance. Carboplatin and miR-21 have an inverse correlation and miR-21 targeted the 3′-UTR of *SMAD7* to prevent its translation. Therefore, the upregulation of miR-21 promotes carboplatin resistance by reducing the level of SMAD7 and enhancing TGFβ receptor signaling mediated NSCLC cell invasion (Lin et al., 2016). LINC01224 expression was upregulated in NSCLC tissues and cell lines compared to adjacent healthy tissues and normal cells. The knockdown of LINC01224 inhibited the NSCLC cells proliferation and invasion *in vitro* and restrained tumor growth *in vivo* model. Furthermore, LINC01224 knockdown significantly enhanced cisplatin sensitivity to NSCLC cells and promoted miR-2467 expression. LINC01224 directly interacted with miR-2467 and partly inverted LINC01224 function in NSCLC cells, such as proliferation, invasion, and cisplatin sensitivity. Therefore, LINC01224 might serve as a novel indicator of cisplatin sensitivity (Xiao et al., 2021).

Similar to Hedgehog, the NOTCH signaling pathway plays a key role in cisplatin resistance. The upregulation of miR-449a reversed cisplatin sensitivity by direct downregulation of the NOTCH1 signaling pathway (You et al., 2014). miR-449a also regulated the expression of lncRNA nuclear enriched abundant transcript 1 (NEAT1), representing the interplay between miRNA and lncRNA. NEAT1 consistently overexpressed in the lymph node of lung cancer metastatic tumors than the primary tumors. Interestingly, overexpression of miR-449a downregulated NEAT1 and reversed back the cisplatin sensitivity. EMT-regulating miRNAs such as miR-98 and miR-200 have been shown to sensitize cisplatin-resistant human LAD cells. The downregulation of miR-98, belongs to let-7 family, and miR-200 have been observed in cisplatin resistance (Ahmad et al., 2013). Tumor suppressor miR-101 plays a crucial role in cisplatin resistance and EMT of NSCLC by targeting Rho-associated coiled-coil-containing protein kinase 2 (ROCK2). ROCK2 is a downstream serine/threonine kinase of the small GTPase Rho. miR-101 complementary binds to the 3′-UTR of ROCK2 mRNA, thereby suppressing the expression of ROCK2. The lower expression of ROCK and overexpression of miR-101 induces cisplatin sensitivity and inhibit EMT *in vitro* lung cancer model. The lower expression of miR-101 correlates with poor survival time by Kaplan-Meier analysis (Ye et al., 2016). LncRNA SNHG1 also upregulates ROCK2 to reduce cisplatin sensitivity in NSCLC cells by targeting miR-101-3p (Wei et al., 2019). miR-101-3p has inverse correlation with SNHG1 or ROCK2 in NSCLC tissues as miR-101-3p can control the expression of SNHG1 by targeting 3′-UTR of SNHG1 mRNA. The downregulation of miR-101-3p promotes cisplatin resistance to NSCLC cells by upregulating SNHG1 and ROCK2. Therefore, the SNHG1/miR101-3p/ROCK2 signaling cascade plays a key role in the cisplatin resistance in NSCLC cells (Wei et al., 2019).

Tumor suppressor miR-218 has inverse correlation with the ‘master switch’ runt-related transcription factor 2 (RUNX2), which controls several genes involved in the development of osteoblasts. The other function of RUNX2 is to modulate angiogenesis *via* cell proliferation, invasion, and tube formation. Upregulation of miR-218 increases cisplatin sensitivity by the downregulation of RUNX2, which enhances apoptosis and the G_0_/S phase cell cycle arrest in NSCLC (Xie et al., 2016). miR-218 is also inversely correlated with EMT transcription factors such as Slug and ZEB2. The upregulation of miR-218 enhanced the chemosensitivity of cisplatin to cells and also obstructed the cell migration and invasion by suppressing Slug and ZEB2 expression by blocking the 3′-UTR regions of *Slug* and *ZEB2* (Shi Z. M. et al., 2017b). LncRNA Growth Arrest-Specific 5 lncRNA (GAS5) generally functions as an anti-oncogene linked to many cancers. A lower level of GAS5 has been observed in NSCLC cells and tissue associated with increasing tumor size and lymphatic metastasis. Besides, the downregulation of GAS5 has been correlated with chemoresistance. Upregulation of GAS5 inhibits the invasion, migration, EMT in NSCLC/DDP cells and also impedes lung tumor growth *in vivo* by inhibiting miR-217/LHPP axis. Phospholysine phosphohistidine inorganic pyrophosphate phosphatase (LHPP) functions as tumor suppressor and interestingly, GAS5 overexpression enhanced LHPP expression whereas miR-217 overexpression reduced LHPP expression. At the same time, silencing of miR-217 increased the expression of GAS5. GAS5 increased the LHPP expression by serving as a ceRNA for miR-217 in cisplatin resistant NSCLC cells. Therefore, GAS5/miR-217/LHPP pathway reduces NSCLC cisplatin resistance and that LHPP may serve as a potential therapeutic target for NSCLC cisplatin resistance (Yang et al., 2021).

Phosphatidylethanolamine-binding protein 4 (PEBP4) is a member of the PEBP family involved in EMT. (PEBP4) is a direct and functional target of miR-15b. miR-15b was significantly upregulated in A549/cisplatin cells compared to parental A549 cells. Also, overexpression of miR-15b was observed in LAD patients treated with cisplatin-based agents in tumor tissues. The upregulation of miR-15b represses the expression of metastasis suppressor PEBP4 and contributes to cisplatin resistance which causes poor prognosis in LAD patients (Zhao Z. et al., 2015b). Therefore, miR-15b-PEBP4 is one of the major pathways involved in EMT regulation and cisplatin resistance in LAD (Zhao Z. et al., 2015b). Raf Kinase Inhibitory Protein (RKIP) is a key regulator in cisplatin resistance in lung cancer. Upregulation of miR-27a induces chemoresistance and EMT by silencing the RKIP in NSCLC. The upregulation of miR-27a and a simultaneous downregulation of RKIP have also been observed in the tumor tissue samples of patients undergoing cisplatin-based therapy (Li et al., 2014).

### 3.8 ncRNAs control resistance to platinum-based agents by regulating other transcription factors

miRNAs also regulate many transcription factors involved in lung cancer chemoresistance as shown in Table 6. Among them, the eukaryotic translation initiation factor (EIF4G2) is regulated by miR-379. Hao *et al* showed that the miR-379 is negatively associated with EIF4G2 in clinical samples of cisplatin-resistant tissues. miR-379 increases cisplatin sensitivity by direct binding to 3 ´UTR of *EIF4G2* but the lower expression of miR-379 induces chemoresistance in A549 cells (Hao et al., 2017). Like miR-379, miR-378 also plays a crucial role in cisplatin resistance by targeting clusterin (CLU), a glycoprotein that regulates the cell cycle, apoptosis, and DNA repair mechanisms. There are two types of CLU: secreted (sCLU) and nuclear (nCLU). The bioinformatics data of Chen *et al* have explained that miR-378 targets the secreted clusterin (sCLU) by partial complement to the 3′ UTR of *sCLU* mRNA (Chen et al., 2016). The clinical samples of advanced LAD proved that miR-378 is a negative regulator of sCLU. The higher expression of miR-378 downregulates the expression of sCLU and increases the cisplatin sensitivity *in vitro* and *in vivo* of lung cancer models. sCLU can also progress chemosensitivity by downregulating Bcl-2, pCas-3, pAkt and pErk1/2. sCLU also can induce chemoresistance by targeting tumor necrosis factor, FAS, TRAIL, and histone deacetylase inhibitors (HDACi). Therefore, CLU is also a vital factor in the regulation of chemoresistance (Chen et al., 2016). Another translation initiation factor, such as eukaryotic translation initiation factor 5A2 (eIF5A2) is also regulated by miR-9 in cisplatin-resistance NSCLC cells. The overexpression of miR-9 increases cisplatin sensitivity by reducing the expression of eIF5A2 (Pan et al., 2018). One of the detoxifying processes of cisplatin is elevating the level of glutathione-S-transferase P1 (GSTP1), GSH, and metallothionein inside the cell. Exogenous miR-513a-3p increases the sensitivity of cisplatin by suppressing the GSTP1 expression (Zhang et al., 2012). GSTP1 is also negatively regulated by another miR-133b by targeting its 3′-untranslated region of *GSTP1.* The overexpression of miR-133b represses the malignant growth, and aggressiveness of cisplatin-resistant NSCLC cells by downregulating the expression of GSTP1 (Lin et al., 2018). miRNAs are not only regulating the sensitivity and chemoresistance involved in monotherapy, they are also involved in doublet chemotherapy. Cao *et al* explained that miR-192 regulates chemoresistance for combined therapy of gemcitabine and cisplatin in A549 cells *via* apoptotic marker Bcl-2 proteins (Cao et al., 2015).

**TABLE 6:** Non-coding RNAs profile alters the expression of others transcription factors involved in cisplatin-resistance.

Non-coding RNAs	Dysregulation	Pathway/target	Sensitivity	Model	References
miR-379	Upregulation	*EIF4G2*	Increase	A549 cells	Hao et al. (2017)
miR-378	Upregulation	sCLU	Increase	*in vitro, in vivo* models of lung cancer	Chen et al. (2016)
miR-9	Upregulation	eIF5A2	Increase	NSCLC	Pan et al. (2018)
miR-133b	Upregulation	GSTP1	Increase	cisplatin-resistant NSCLC	Lin et al. (2018)
miR-24-3p	Downregulation	ATG4A	Decrease	SCLC	Pan et al. (2018)

## 4 Conclusion

**FIGURE 4:**
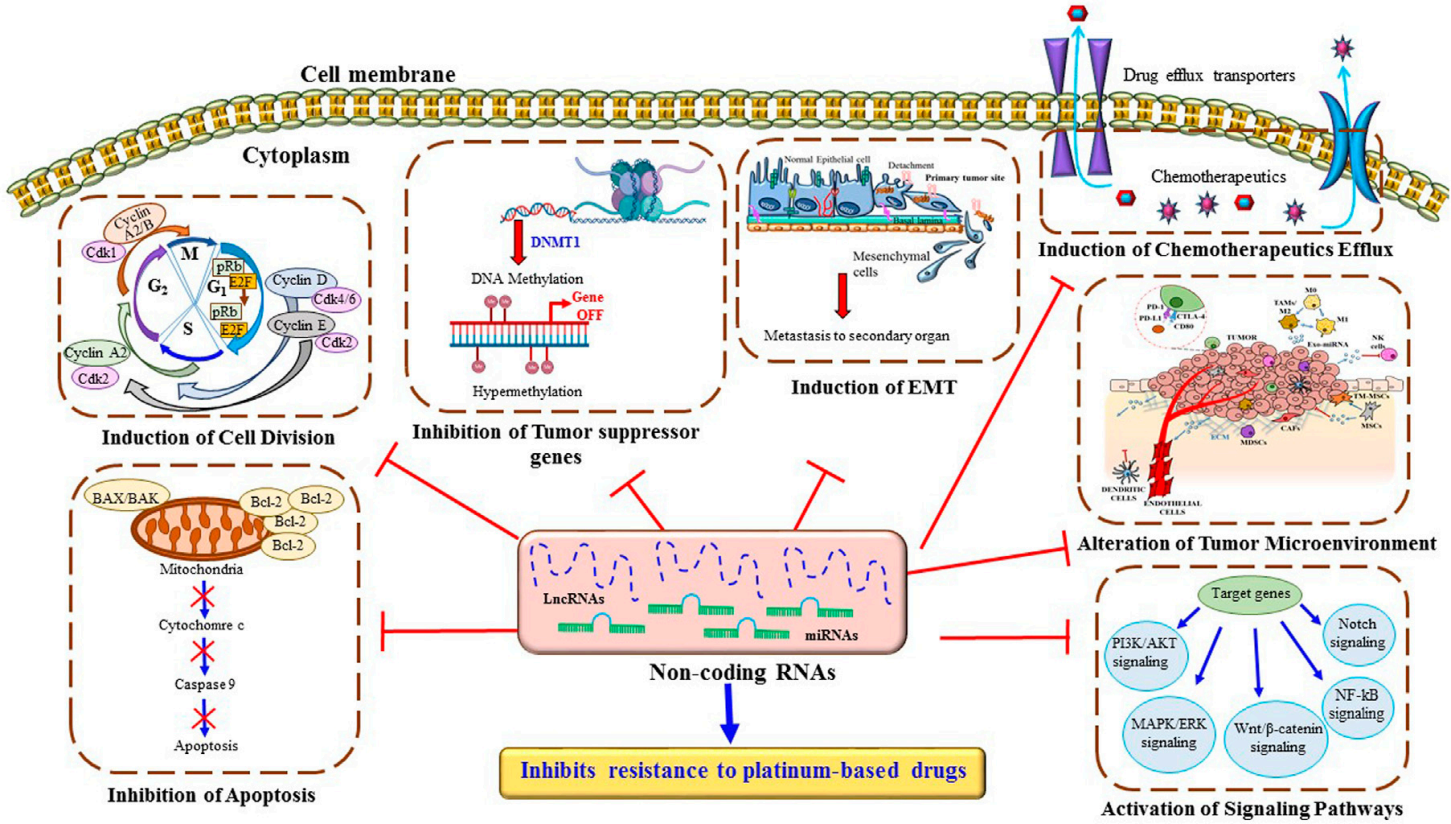
Major mechanisms through ncRNAs inhibit resistance to platinum-based drugs in lung cancer cells. ncRNAs regulate multiple cellular mechanisms such as drug efflux, DNA repair pathways, intrinsic and extrinsic cell death programs, cell cycle, metastasis, and several signaling pathways, which contribute to platinum-based drug resistance in lung cancer cells.

Platinum-based antitumor agents have been shown to have significant efficacy in clinical trials against cancer therapy. Despite years of research and broad clinical trial, chemoresistance to platinum drugs remains a big challenge in lung cancer therapy. Several basic to advanced mechanisms have been identified in platinum-based drug resistance. Among, alterations in detoxification enzymes, TME, autophagy, hypoxia, apoptotic pathway, DNA repair mechanism and epigenetic modifications play a significant role Figure 4. Recent scientific research has identified some therapeutic approaches to overcome chemoresistance in lung cancer. Among them, ncRNA can be an innovative target to reverse the sensitivity of platinum-based drugs by suppressing the over activity of drug efflux transporters, and enhancing the expression of cell-cycle-related proteins and pro-apoptotic factors. Both miRNAs and lncRNAs can alter several signaling pathways related to EMT, apoptosis, DNA repair mechanisms, and TME. Therefore, ncRNAs can be considered as a therapeutic target to enhance the anti-cancer effect of platinum-based agents. Besides chemoresistance, the unsatisfactory prognosis tools of NSCLC are one of the major reasons behind the high mortality rate due to lung cancer. Therefore, there is a requirement to find more powerful prognostic regimes for better therapeutic outcomes for lung cancer patients. In our recent study, we have compiled a list of ncRNAs that can overcome platinum-based drug resistance in lung cancer cells. Some of them can also be used as diagnostic and prognostic markers to identify the stage of chemoresistance. For example, serum miRNA 125b can be used as a diagnostic or prognostic biomarker for advanced NSCLC patients under cisplatin-based chemotherapy. Because clinical samples of patients with advanced NSCLC have shown overexpression of miR-125b causes remarkable inhibition of cisplatin-induced apoptosis, thereby enhancing cisplatin resistance. Besides ncRNAs, genes such as TP53 and MDM2 or the expression of DNA repair enzymes like ERCC1 can be used as a prognostic and predictive factor in lung cancer patients to regulate the sensitivity of cisplatin. In the present study, we have described in detail of some of the miRNAs or lncRNAs that can be used as therapeutic tools to alter the expression of ERCC1 and reverse back the cisplatin sensitivity in lung cancer cells. Therefore, ncRNAs can be an important advanced therapeutic choice to improve the traditional therapeutic approaches against lung cancer. Overall, the novelty of our study, we have illustrated the new therapeutic approaches of ncRNAs to overcome platinum-based drug resistance in lung cancer and also portrayed ncRNAs as the diagnostic and prognostic biomarkers to identify the advancement of chemoresistance.
